# Characteristics of Cognitive Impairment and Their Relationship With Total Cerebral Small Vascular Disease Score in Parkinson’s Disease

**DOI:** 10.3389/fnagi.2022.884506

**Published:** 2022-07-07

**Authors:** Miaomiao Hou, Xiaojun Hou, Yiqing Qiu, Jiali Wang, Mingyang Zhang, Xiaowei Mao, Xi Wu

**Affiliations:** ^1^Department of Neurology, Xinhua Hospital Affiliated to Shanghai Jiao Tong University School of Medicine, Shanghai, China; ^2^Department of Neurology, The First Affiliated Hospital of Naval Medical University, Shanghai, China; ^3^Department of Neurosurgery, The First Affiliated Hospital of Naval Medical University, Shanghai, China; ^4^Department of Chemistry, University of Utah, Salt Lake City, UT, United States

**Keywords:** Parkinson’s disease, cerebral small vascular disease, cognitive impairment, CSVD burden, MRI

## Abstract

**Background:**

This study aimed to investigate the characteristics of cognitive dysfunctions and their relationship with total cerebral small vascular disease (CSVD) in Parkinson’s disease (PD).

**Methods:**

A total of 174 idiopathic PD patients who underwent brain magnetic resonance imaging (MRI) were recruited. Demographic information, vascular disease risk factors, motor function (MDS-UPDRS III score), and cognitive level (MoCA, MMSE) were collected for these patients. The total CSVD burden was scored based on lacunes, enlarged perivascular spaces (EPVS), high-grade white matter hyperintensities (WMH), and cerebral microbleeds (CMBs) for each subject.

**Results:**

Cognitive scores on MoCA for language, delayed recall, and orientation were significantly reduced in PD patients with CSVD burden ≥ 1 than in those with CSVD burden = 0. Educational level, PDQ 39, and CSVD burden were significantly associated with MoCA scores in individuals with PD. For the whole group, the full model accounted for 33.6% variation in total MoCA scores. In which, CSVD burden explained 2.7% of the results, and the detection of lacunes, WMH, EPVS, and strictly lobar CMBs were significantly correlated with MoCA scores. The stability of the outcomes was confirmed by sensitivity analysis.

**Conclusion:**

CSVD can independently contribute to cognitive decline in PD and cause damage in specific cognitive domains. Promoting neurovascular health may help preserve cognitive functions in PD.

## Introduction

Parkinson’s disease (PD), a progressive motor and cognitive degeneration disease, is clinically characterized by cardinal motor dysfunction symptoms, such as rigidity, resting tremor, bradykinesia, and gait/postural instability, in addition to several non-motor deficits primarily including anxiety, cognitive decline, depression, and gastrointestinal dysfunction. Among them, the prevalence of cognitive impairment has been estimated up to 20–35% in the early stage of PD (Svenningsson et al., 2012; Hu et al., 2014; Pfeiffer et al., 2014). Patients with PD gradually progress to PD and related dementia (PDD) at a rate of about 10% every year, and about 75% of PD patients, mostly with an advanced disease condition, suffer from PDD, as the past 10 years of data has shown (Aarsland and Kurz, 2010). However, the underlying disease-causing mechanisms of cognitive decline in PD patients have not been fully revealed. Notably, dozens of etiopathological factors are considered to play a role in the PD mechanism, of which vascular pathology is thought to be a major culprit.

*In vivo* imaging studies showed the frequent presence of white matter hyperintensity (WMH), one of the key diagnostic indicators of cerebral small vessel disease (CSVD), was found in 30–55% of PD patients, which was more prevalent than in age-matched normal subjects (Sohn and Kim, 1998; Lee et al., 2009). Additionally, the white matter lesion burden may double the risk of PDD development (Choi et al., 2010). CSVD is characterized by pathological hallmarks like WMH, perivascular space (PVS), microbleeds, and lacune on MRI, and can lead to emotional disorders, cognitive impairment, and gait dysfunction (Wardlaw et al., 2013). Not only concerning clinical symptoms, PD and CSVD also have similarities in mode of pathogenesis, including cerebral microangiopathy, neuroinflammatory injury, and impaired blood-brain barrier (Sweeney et al., 2018; Wardlaw et al., 2019). Because of these overlapping pathomechanisms, PD patients comorbid with CSVD exhibit worse symptoms than patients with PD only.

Although earlier studies exploited one or two widely used markers of CSVD, the CSVD burden score can better assess the overall disease severity by integrating four CSVD markers. To date, only a couple of studies by Shibata et al. (2019) and Chen et al. (2021) have applied CSVD burden scores in their analyses of cognitive impairment, indicating a significant correlation between them. Therefore, this retrospective study was designed to evaluate the possibility of utilizing the CSVD burden score as the independent predictor of PDD onset in the clinical setting and further explore the cognitive deficit characteristics in PD patients comorbid with CSVD.

## Materials and Methods

### Subject Selection

A total of 174 study participants diagnosed with idiopathic PD were recruited between January 2021 and February 2022 from the outpatient department of Neurology and Neurosurgery, the first affiliated hospital of Navy Medical University under the protocol approved by the hospital’s ethics committee. Written informed consents were collected from all participants. The finally recruited patients all met the clinical diagnostic criteria of the Movement Disorder Society (MDS) and reported a positive response to levodopa therapy. The exclusion criteria for the subjects were: (1) diagnosed with toxin-, pathogen- and drug-induced-parkinsonism syndrome or other degenerative forms of parkinsonism including progressive supranuclear palsy (PSP), corticobasal ganglionic degeneration (CGD), and multiple-system atrophy (MSA); (2) history of stroke, moderate-to-severe head trauma, brain tumor, hydrocephalus or psychopathological disorders with 14-item Hamilton Anxiety Rating Scale (HAM-A) ≥ 14 or 24-item Hamilton Depression Rating Scale (HAM-D) ≥ 20; and (3) inability to coordinate or communicate with clinicians.

### Procedures and Measures

#### Clinical Assessment

Socio-demographic profiles of the recruited subjects, including age, gender, smoking and drinking habits, education level, medical history (ischemic stroke, diabetes, and hypertension), and disease duration, were collected and carefully reviewed. The diagnosis criteria for hypertension and diabetes were based on the 2020 International Society of Hypertension global hypertension practice guidelines (Unger et al., 2020) and Standards of Medical Care in Diabetes-2020 (Buse et al., 2020), respectively. According to the definition of smoking status in the National Health Interview Survey, an adult who has smoked 100 cigarettes in his or her lifetime, no matter whether he or she still smokes or had quit smoking at the time of the interview was defined as current or previous smoking. Blood pressure reading and serum lipid profiling results (triglyceride, total cholesterol, low-density lipoprotein, high-density lipoprotein) were recorded on admission. The definition of hyperlipidemia was based on 2016 Chinese guidelines for the management of dyslipidemia in adults (Joint committee issued Chinese guideline for the management of dyslipidemia in adults, 2016). Dyslipidemia diagnosis were made when: TC ≥ 6.2 mmol/L (240 mg/dl) or TG ≥ 2.3 mmol/L (200 mg/dl) or HDL-C < 1.0 mmol/L (40 mg/dl) or LDL-C ≥ 4.1 mmol/L (160 mg/dl). The MDS-Unified PD Rating Scale part III (MDS-UPDRS III), and Hoehn–Yahr staging scores were used to assess the disease severity in each patient both in the on and off-medication state, while cognitive performance evaluation was carried out using Mini-Mental State Examination (MMSE) as well as Montreal Cognitive Assessment (MoCA) scales in the on-medication state. The psychopathological screening was done by a 14-item HAM-A and a 24-item HAM-D. The quality of life was assessed by PD questionnaire 39 (PDQ39).

#### Magnetic Resonance Imaging Data Acquisition and Evaluation

MRI data were acquired following the imaging sequences- T1-weighted (T1-W), T2-W, fluid-attenuated inversion recovery imaging (FLAIR), and susceptibility-weighted imaging (SWI) from two 3T MRI devices in our hospital. Image readers were blinded to patient information and performed image reviews independently. M.M. Hou and X.W. Mao evaluated MR scan images based on the CSVD markers, following the guideline standards for reporting vascular changes on neuroimaging (STRIVE) (Wardlaw et al., 2013). WMH was scored on the Fazekas scale (Fazekas et al., 1993), and the basal ganglia PVS (BG-PVS) was rated on a semi-quantitative rating scale (Doubal et al., 2010). Due to the well-established relationship with the pathology of CSVD, the topographic distribution of CMBs was divided into CMBs in strictly lobar regions which are related to CAA, and CMBs in the deep and/or infratentorial (D/I) areas which are associated with hypertensive arteriopathy (Gregoire et al., 2009; Yakushiji et al., 2011, 2019). The topographic distribution of lacunes was defined as similar to that used for CMBs, that is, lacunes in the deep areas such as the BG, thalamus, internal capsule, and pons and lacunes in the lobar areas such as centrum semiovale (Pasi et al., 2017). The inter-rater agreement rate and the test-retest reliability were tested by the κ coefficient. The intra- (κ = 0.565) and the inter-rater reliability (κ = 0.679) regarding CSVD burden score were good.

The CSVD burden score was rated with one point allocated to each of the following MRI parameters: severe WMH (confluent deep WMH Fazekas 2–3 or irregular periventricular WMH Fazekas 3), presence of ≥ 1 lacunes, ≥ 1 microbleeds in the deep or lobar cerebral region, and moderate-to-severe BG-PVS (semi-quantitative rating 2–4), with total scores ranging from 0 to 4 points.

### Statistical Analysis

Continuous variables were presented as mean ± standard deviation (SD) and categorical variables as median (quartiles). The etiological risk factors were correlated with cognitive impairment in PD using Spearman’s correlation analysis. The significance of the result was considered at *P* < 0.05 (two-sided). An unadjusted multivariate linear regression model with the forward stepwise method was applied to assess the association of MoCA scores with the risk factors filtered by Spearman’s correlation. As for propensity score matching (PSM), a binary logistic regression model was conducted according to outcome variables (CSVD = 0 vs. ≥ 1) with the forward stepwise method. The variables (age, education, and smoking) were selected as covariates for PSM. Finally, 57 patients with CSVD = 0 and 50 patients with CSVD ≥ 1 were successfully matched and included in the subsequent analyses. The comparisons of MoCA subscores between CSVD 0 and ≥ 1 were performed using the two-sample *t*-test after PSM. SPSS (version 25.0) was used for all the above statistical analyses. Sensitivity analysis was conducted to investigate the stability of the outcomes. To avoid MoCA scores not conforming to a normal distribution and to make relative comparisons across cognitive domains, MoCA scores and subscores were converted into Z-scores based on the mean and standard deviation of the total sample at baseline. The multivariate linear regression with Z-scores of MoCA as the dependent variable and the comparison of Z-scores of MoCA subscores between CSVD 0 and ≥ 1 after PSM by two-sample *t*-test were performed to check whether the results were consistent with those before.

## Results

**FIGURE 1:**
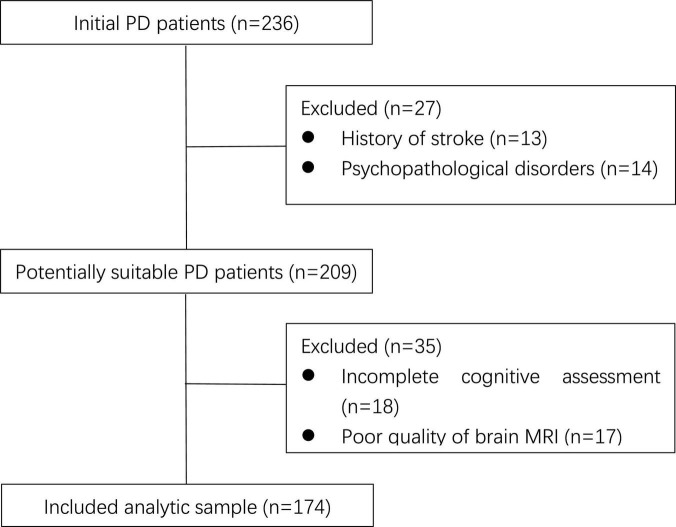
Flowchart of patient enrollment. PD, Parkinson’s disease; MRI, magnetic resonance imaging.

At first, there were 236 PD patients recorded in our database. According to the exclusion criteria, 27 patients were excluded due to stroke history and serious mood disorders, 18 patients failed to complete cognitive assessment due to communication difficulties or other reasons, and 17 patients were excluded because their brain MRI were not clear enough to perform CSVD score. The flowchart is shown in Figure 1.

174 eligible PD patients were finally recruited with a mean age of 62.87 ± 9.33 years. In this group, there were 78 female subjects (44.8%). Lacunes were present in 14.9%, PVWMH, and DWMH in 52.3 and 58.0%, respectively, BG-PVS in 55.2%, CSO-PVS in 72.9%, and CMBs in 7.5% patients.

Spearman’s correlations indicated that the MoCA score was positively correlated with education, smoking habit, and MMSE. Age, female gender, hyperlipidemia, Hoehn-Yahr staging, MDS-UPDRS III, HAM-D, HAM-A, PDQ 39, NMSS, presence of lacune (lobar and deep), PVWMH, DWMH, BG-PVS, strictly lobar CMBs, and CSVD total burden score were negatively correlated with MoCA score (Table 1).

**TABLE 1:** Demographic profiles of PD patients and Spearman’s correlation with MoCA score.

Characteristics	Total sample (*n* = 174)	*r*	*P*
Age (years), mean ± SD	62.87 ± 9.33	–0.198	0.015
Gender, *n* (female) (%)	78 (44.8%)	–0.160	0.050
Education (years), mean ± SD	9.27 ± 4.38	0.349	<0.001
**Medical history**			
Hypertension, *n* (%)	45 (25.9%)	0.085	0.315
Diabetes, *n* (%)	20 (11.5%)	–0.040	0.634
Hyperlipidemia, *n* (%)	24 (13.8%)	–0.266	0.003
Current or previous smoking, *n* (%)	53 (30.5%)	0.235	0.005
Supine systolic blood pressure (mmHg), mean ± SD	125.30 ± 16.12	0.056	0.502
Supine diastolic blood pressure (mmHg), mean ± SD	75.42 ± 11.53	0.083	0.318
**Disease profile**			
Disease duration (years), mean ± SD	10.12 ± 6.17	–0.131	0.110
Hoehn-Yahr staging, median (IQR)	3 (3.4)	–0.253	0.002
MDS-UPDRS III			
Off, mean ± SD	59.82 ± 16.31	–0.259	0.001
On, mean ± SD	31.11 ± 15.94	–0.191	0.019
Levodopa equivalent daily dosage at admission (mg), mean ± SD	759.42 ± 424.21	0.057	0.484
HAMD, mean ± SD	13.57 ± 5.57	–0.269	<0.001
HAMA, mean ± SD	10.80 ± 4.50	–0.358	<0.001
MoCA, mean ± SD	22.51 ± 4.70		
MMSE, mean ± SD	25.38 ± 4.23	0.765	<0.001
PDQ-39, mean ± SD	65.31 ± 22.73	–0.364	<0.001
NMSS, mean ± SD	17.91 ± 3.68	–0.339	<0.001
**MRI features**			
Lacunes presence, *n* (%)	26 (14.9%)	–0.173	0.037
Deep lacunes, *n* (%)	24 (13.8%)	–0.169	0.041
Lobar lacunes, *n* (%)	6 (3.4%)	–0.214	0.010
2-3 DWMH, *n* (%)	14 (8.0%)	–0.351	0.001
3 PVWMH, *n* (%)	26 (14.9%)	–0.410	0.001
2-4 BG-PVS, *n* (%)	38 (21.8%)	–0.207	0.006
2-4 CSO-PVS, *n* (%)	27 (15.5%)	–0.091	0.135
CMBs presence, *n* (%)	13 (7.5%)	–0.153	0.065
Strictly lobar CMBs, *n* (%)	5 (2.9%)	–0.165	0.047
D/I or mixed CMBs, *n* (%)	8 (4.6%)	–0.024	0.775
CSVD burden, median (IQR)	0 (0.1)	–0.307	<0.001

*PD, Parkinson’s disease; MoCA, Montreal Cognitive Assessment; MDS-UPDRS III, movement disorder society united Parkinson’s disease rating scale part III; HAMA, Hamilton Anxiety Rating Scale; HAMD, Hamilton Depression Rating Scale; MMSE, Mini-mental State Examination; PDQ39, Parkinson’s disease questionnaire 39; NMSS, non-motor symptoms scale; MRI, magnetic resonance imaging; WMH, white matter hyperintensity; EPVS, enlarged perivascular spaces; CMBs, cerebral microbleeds; CSVD, cerebral small vessel disease; SD, standard deviation; DWMH, deep white matter hyperintensity; PVWMH, periventricular white matter hyperintensity; BG, basal ganglia; CSO, centrum semiovale; PVS, perivascular spaces; D/I, deep and/or infratentorial; IQR, interquartile range.*

Multivariate linear regression further demonstrated that MoCA score was significantly associated with education, PDQ 39 score, and CSVD burden, accounting for 33.6% variance in MoCA scores (*P* < 0.05). In which, CSVD burden explained 2.7% of the results (Table 2 and Supplementary Table 1). Multicollinearity analysis confirmed that the contribution of each aspect was independent of the regression model. The condition index was < 10, and the variance inflation factor (VIF) was < 2.

**TABLE 2:** Multivariate linear regression analysis of factors associated with MoCA scores.

Model	Fitting effect	Coefficient β (95%CI)	*P*	VIF
Constant	*R* = 0.592	23.239 (20.370∼26.109)	0.000	
Education	*R*^2^ = 0.351	0.441 (0.271∼0.661)	0.000	1.022
PDQ39	Adjusted *R*^2^ = 0.336	−0.071 (−0.103∼−0.039)	0.000	1.082
CSVD burden		−1.172 (−2.100∼−0.245)	0.014	1.089

*MoCA, Montreal Cognitive Assessment; PDQ39, Parkinson’s disease questionnaire 39; CSVD, cerebral small vessel disease, VIF, variance inflation factor.*

Clinical characteristics between CSVD = 0 and ≥ 1 before and after PSM were shown in Table 3. Before PSM, patients with CSVD ≥ 1 were older, had a more extensive history of smoking, and had a shorter educational period. The PSM procedure has successfully addressed the covariate imbalance. In cognitive function analysis, patients with CSVD burden ≥ 1 displayed significantly lower MoCA total score as well as subscores of language, delayed recall, and orientation than those in the CSVD burden = 0 group (Table 4).

**TABLE 3:** Clinical characteristics between CSVD = 0 and ≥ 1 before and after propensity score matching.

	Before matching (*n* = 174)			After matching (*n* = 107)		
	CSVD = 0 (*n* = 96)	CSVD ≥ 1 (*n* = 78)	*P*	CSVD = 0 (*n* = 57)	CSVD ≥ 1 (*n* = 50)	*P*
Age (years), mean ± SD	60.87 ± 9.65	65.37 ± 8.31	0.001	63.98 ± 8.06	64.17 ± 8.27	0.898
Gender, *n* (female) (%)	45 (46.9%)	33 (42.3%)	0.547	30 (52.6%)	21 (42.0%)	0.219
Education (years), mean ± SD	10.16 ± 4.04	8.17 ± 4.57	0.002	8.92 ± 3.93	8.69 ± 4.16	0.746
**Medical history**						
Hypertension, *n* (%)	22 (22.9%)	23 (29.5%)	0.325	16 (28.1%)	15 (30.0%)	0.700
Diabetes, *n* (%)	7 (7.3%)	13 (16.7%)	0.054	5 (8.8%)	8 (16.0%)	0.286
Hyperlipidemia, *n* (%)	10 (10.4%)	14 (17.9%)	0.152	12 (21.1%)	9 (18%)	0.741
Current or previous smoking, *n* (%)	23 (24.0%)	30 (38.5%)	0.039	17 (29.8%)	16 (32.0%)	0.704
Supine systolic blood pressure (mmHg), mean ± SD	123.43 ± 16.88	127.53 ± 14.96	0.098	125.87 ± 16.93	126.70 ± 15.23	0.772
Supine diastolic blood pressure (mmHg), mean ± SD	74.23 ± 11.07	76.83 ± 11.99	0.142	76.00 ± 10.73	75.03 ± 11.53	0.625
**Disease profile**						
Disease duration (years), mean ± SD	9.41 ± 3.87	10.99 ± 8.12	0.117	9.49 ± 3.95	10.87 ± 8.69	0.247
Hoehn-Yahr staging, median (IQR)	3 (3.4)	3 (3.4)	0.194	3 (3.4)	3 (3.4)	0.161
MDS-UPDRS III						
Off, mean ± SD	56.97 ± 16.73	63.33 ± 15.16	0.010	59.42 ± 15.98	66.18 ± 14.26	0.012
On, mean ± SD	28.14 ± 15.99	34.76 ± 15.20	0.006	30.08 ± 16.14	35.03 ± 14.74	0.070
Levodopa equivalent daily dosage at admission (mg), mean ± SD	796.39 ± 395.18	714.02 ± 455.80	0.201	804.70 ± 423.53	718.08 ± 433.69	0.251
HAMD, mean ± SD	13.00 ± 5.11	14.27 ± 6.05	0.134	12.34 ± 4.74	15.28 ± 5.91	0.002
HAMA, mean ± SD	10.39 ± 4.09	11.30 ± 4.93	0.182	9.58 ± 3.98	11.86 ± 4.73	0.004
MoCA, mean ± SD	23.82 ± 3.54	20.57 ± 5.50	< 0.001	23.32 ± 4.17	20.46 ± 5.73	0.004
MMSE, mean ± SD	26.44 ± 3.43	24.08 ± 4.74	< 0.001	25.77 ± 3.97	24.28 ± 4.11	0.037
PDQ-39, mean ± SD	62.84 ± 20.32	68.35 ± 25.19	0.121	61.54 ± 18.84	71.73 ± 24.06	0.009
NMSS, mean ± SD	17.66 ± 3.44	18.22 ± 3.97	0.322	17.85 ± 3.35	18.74 ± 3.30	0.128

*CSVD, cerebral small vessel disease; MoCA, Montreal Cognitive Assessment; MDS-UPDRS III, movement disorder society united Parkinson’s disease rating scale part III; HAMA, Hamilton Anxiety Rating Scale; HAMD, Hamilton Depression Rating Scale; MMSE, Mini-mental State Examination; PDQ39, Parkinson’s disease questionnaire 39; NMSS, non-motor symptoms scale; SD, standard deviation; IQR, interquartile range.*

**TABLE 4:** Comparison of MoCA subscores between CSVD = 0 and ≥ 1 after propensity score matching.

	CSVD = 0 *N* = 57	CSVD ≥ 1 *N* = 50	*P*
Total score	23.32 ± 4.17	20.46 ± 5.73	0.004
Visuospatial/executive	2.37 ± 1.63	1.86 ± 1.70	0.118
Naming	2.88 ± 0.38	2.76 ± 0.56	0.213
Attention	5.49 ± 0.89	5.36 ± 0.92	0.455
Language	2.58 ± 0.68	2.24 ± 0.96	0.040
Abstraction	1.26 ± 0.64	1.22 ± 0.65	0.730
Delayed recall	2.86 ± 1.43	2.18 ± 1.38	0.014
Orientation	5.74 ± 0.81	5.22 ± 1.27	0.015

*MoCA, Montreal Cognitive Assessment; CSVD, cerebral small vessel disease.*

In sensitivity analysis, the statistical results with Z-scores of MoCA scores and subscores were consistent with those before (Supplementary Tables 2, 3).

## Discussion

This study aimed to examine the pathoclinical association of CSVD with cognitive deficits in PD patients. The CSVD score was independently correlated with the MoCA score. Compared with PD patients with a CSVD burden score of 0, patients with a CSVD burden score ≥ 1 were significantly affected in multiple cognitive domains related to language, memory recall, and orientation in MoCA analysis, suggesting that CSVD comorbidity may contribute to PD aggressiveness and need to be appropriately managed.

We found that a higher CSVD burden could be independently associated with severely impaired cognition in PD patients, which was consistent with earlier findings. A large prospective longitudinal study involving early stage PD patients revealed that cognitive dysfunction combined with unsatisfactory response to long-term levodopa treatment was prevalent in patients comorbid with vascular diseases. Overlapping cerebrovascular and PD syndromes have been categorized in a distinct disease phenotype, motor-cognitive risk syndrome, characterized by slow gait and cognitive complaints. Multiple CSVD markers can impose synergistic effects on cognitive impairment progression. There could be several mechanisms underlying the interaction between CSVD and PD cognition. It has been confirmed that CSVD lesions induce cortical atrophy and disrupt the integrity of brain signaling networks, including the monoamine neurotransmitter system, limbic system, and default network, resulting in emotional disturbance and cognitive dysfunction (Foo et al., 2016; Borgonovo et al., 2017). The crosstalk between neurodegenerative processes (e.g., Lewy body deposition) in PD and cerebrovascular dysfunction remains unclear and requires further study.

Our results also highlighted the important roles of lacunes, WMH, EPVS, and strictly lobar CMBs as CSVD markers in assessing the cognitive dysfunction of PD patients. Several studies have confirmed that WMH is closely related to motor and cognitive impairment in PD patients. Sunwoo et al. (2014) has shown that WMH burden is an important predictor of cognitive decline and progression to dementia in PD patients. Further, Malek et al. (2016) have pointed out that PD patients with WMH at the beginning of the course of the disease are more likely to have rapid cognitive decline than patients without WMH. WMH in different parts of the brain is related to the impairment in different cognitive domains. Frontal WMH may damage the cortical basal ganglia circuit, which was related to the decline of executive function and speech fluency in Parkinson’s disease (Theilmann et al., 2013), while WMH in the frontotemporal region may damage the Papez circuit and frontal subcortical circuit, which was associated with the decline of attention, visuospatial memory and learning ability (Mak et al., 2015). Enlarged PVS (EPVS) has been shown to be linked to pathological features of Alzheimer’s disease (AD) (Ramirez et al., 2016). Furthermore, a meta-analysis of 5 population-based studies also confirmed no association between EPVS and cognitive dysfunction in the general population (Hilal et al., 2018). To date, only one study has reported that the increase of EPVS may be related to PD cognitive impairment, supporting our findings (Chen et al., 2021). Maclullich et al. (2004) have also suggested that EPVS may reduce the responsiveness of PD patients to levodopa, indicating that the diagnosis of EPVS may affect the prognosis of PD patients. Few studies were focusing on the effect of lacunes on cognitive impairment in PD patients, e.g., a multicenter prospective longitudinal cohort study reported that 23.7% of the 882 PD patients had lacunes (Ham et al., 2016), suggesting that not all acute infarcts damage cognitive domains in the brain. It is speculated that cognitive impairment may be associated with lacunes in the thalamus, basal ganglia, and frontotemporal lobe, resulting in impairment of executive function, memory, information processing, and overall cognitive function by affecting the prefrontal hypothalamic circuit (Reed et al., 2004). Studies have shown that the detection rate of cerebral microbleeds in PD patients with dementia is as high as 26.1–36.6% (Ham et al., 2014; Kim et al., 2015), compared to that in PD patients without cognitive impairment and in a healthy population. However, another study has shown there is no significant correlation between microbleeds and cognition in patients with primary PD (Ham et al., 2016). So, there is no definite conclusion at present, and further large sample clinical observations and follow-ups are needed.

Interestingly, we found a significantly positive correlation between current or previous smoking and MoCA score. Unfortunately, we did not record the number of the smoking pack years, smoking cessation time, etc., which affected further detailed analysis. In recent decades, more than 40 epidemiological studies have proved an inverse association between smoking and the prevalence of PD (Quik, 2004). Nicotine may help improve some symptoms of PD, such as dyskinesia and memory impairments (Quik et al., 2008). In fact, both *in vitro* and *in vivo* studies have shown that nicotine played a neuroprotective role in PD through its pro-survival effects on dopaminergic neurons and may serve as a novel therapeutic approach for this population (Barreto et al., 2014). However, the relationship between smoking and cognitive impairment in PD patients has been controversial. For example, a prospective study over an 8-year period did not show significant differences in the progression of parkinsonism as well as cognitive impairment in smoking and non-smoking PD patients (Alves et al., 2004). Doiron et al. (2017) showed that smoking history was associated with global cognitive impairment in PD even in patients who had quit smoking. It is undeniable that smoking is the main risk factor for respiratory and cardiocerebrovascular diseases, and our sample excluded patients with stroke history, resulting in selection bias. Based on our findings, it will be our next study topic to verify the relationship between smoking and Parkinson’s disease cognition.

As for the research field in PD cognitive function, robust evidence has demonstrated that compared with age-matched groups without PD, patients with PD have more significant cognitive impairment in many cognitive domains, including executive, attentional and visuospatial domains, along with memory (Svenningsson et al., 2012). A multicenter study for the validity of MoCA in the detection of MCI and dementia in PD found that patients with the MCI or PDD had significantly lower visuospatial/executive, attention, language, delayed recall, and orientation subscores (Hoops et al., 2009). Regarding the performance of individual cognitive domains in our study, we found that CSVD burden could affect language, memory recall, and thought orientation. Banerjee et al. (2018) have found that frontal and visuospatial tasks can be influenced by total CSVD scores in a memory clinic population. Also, Liang et al. (2019) have confirmed that total CSVD burden is correlated with reduced MMSE subscores for thought orientation, word recall, and calculation in a post-stroke population. Li et al. (2021) have also shown that memory, executive function, speed, and motor control were correlated with a higher total CSVD burden in a propensity score-matched case-control study. The overlap of affected cognitive domains in PD and CSVD may be due to the impairment of similar signaling networks despite the involvement of multiple pathogeneses. However, the wide variety of the profile and rate of cognitive decline among individuals with PD requires careful observation and long-term follow-up. No current publications to our knowledge had investigate the impact of CSVD burden on cognitive actions in PD patients. Hence, early stage multidimensional neuropsychiatric assessments and stratified analysis of PD subtype are critical to screen for the risk of cognitive impairment in PD patients with CSVD and choose targeted treatment methods.

The main strength of our study is the good generalizability of the conclusion. Outpatients with PD are mostly mild CSVD burden, and our research represented this population, which is the intervention target for future CSVD management. Other strengths include evaluation using the recently reported CSVD burden score with details on the locations of CMBs and lacunes, as well as the PSM approach to addressing the covariate imbalance in comparison between groups. The limitations of our study are as follows: first, the MMSE and MoCA might not have sufficient sensitivity for detecting mild cognitive changes in an individual domain in PD. In-depth neuropsychological correlation analyses between CSVD and the specific cognitive domain are required; second, due to the distribution characteristics of CSVD burden score in this sample, we merely use 1 as the cutoff of CSVD burden to carry out a binary logistic regression. It is necessary to expand the sample size and consciously collect patients with higher CSVD scores for further analysis; third, APOE genotypes were not tested in these patients, which yielded confounding bias due to its correlation with both CSVD markers and cognitive impairment. Last but not the least, the cross-sectional nature of our study might not suffice to confirm causal relationships between outcomes and independent variables. Future longitudinal studies are required to examine the impact of CSVD on PD outcomes.

## Conclusion

Cognitive impairment and its advanced stage (dementia) are considered the consequences of the progressive neurodegenerative processes in PD, which not only seriously affect the quality of life and prognosis, but also bring a heavy burden on the patient’s family and society. Although the research on PD-associated cognitive impairment and CSVD is gradually increasing, its pathological mechanism is still unclear. There was no clinical research on whether the intervention of CSVD could be conducive to improving PD cognitive impairment and delaying the disease progression. Therefore, in the future, large-scale prospective studies and relevant basic experiments are needed to clarify the relationship and specific mechanism between CSVD and PD cognitive impairment to provide clues for better management of this disease.

## Data Availability Statement

The raw data supporting the conclusions of this article will be made available by the authors, without undue reservation.

## Ethics Statement

The studies involving human participants were reviewed and approved by the Ethics Committee of the First Affiliated Hospital of Naval Medical University. The patients/participants provided their written informed consent to participate in this study.

## Author Contributions

XW and XM designed the research. XH and JW examined the patients and collected the data. MH and YQ performed the statistical analyses and prepared the manuscript. MZ checked statistical methods and ensured language quality. All authors critically reviewed the content, approved the final version of this article, and contributed to this study.

## Conflict of Interest

The authors declare that the research was conducted in the absence of any commercial or financial relationships that could be construed as a potential conflict of interest.

## Publisher’s Note

All claims expressed in this article are solely those of the authors and do not necessarily represent those of their affiliated organizations, or those of the publisher, the editors and the reviewers. Any product that may be evaluated in this article, or claim that may be made by its manufacturer, is not guaranteed or endorsed by the publisher.
