# Supplementary material for: Characteristics of Cognitive Impairment and Their Relationship With Total Cerebral Small Vascular Disease Score in Parkinson’s Disease
Source: Front Aging Neurosci. 2022 Jul 7;14:884506. doi: 10.3389/fnagi.2022.884506 (PMC9301002; doi:10.3389/fnagi.2022.884506)
Supplement: Supplementary file 1 [file Table_1.docx]

**Supplementary table 1**. Model summary of multivariate linear regression analysis of factors associated with MoCA scores

| Model | R | R^2^ | Adjusted R^2^ | F | *P* |
| --- | --- | --- | --- | --- | --- |
| 1 | 0.426 | 0.182 | 0.175 | 29.07 | 0.000 |
| 2 | 0.565 | 0.319 | 0.309 | 30.47 | 0.000 |
| 3 | 0.592 | 0.351 | 0.336 | 23.22 | 0.000 |

Model 1 predictor: Education; Model 2 predictors: Education, PDQ39; Model 3 predictors: Education, PDQ39, CSVD burden.

Abbreviations: MoCA, Montreal Cognitive Assessment; PDQ39, Parkinson’s disease questionnaire 39; CSVD, cerebral small vessel disease.
