# Supplementary material for: Characteristics of Cognitive Impairment and Their Relationship With Total Cerebral Small Vascular Disease Score in Parkinson’s Disease
Source: Front Aging Neurosci. 2022 Jul 7;14:884506. doi: 10.3389/fnagi.2022.884506 (PMC9301002; doi:10.3389/fnagi.2022.884506)
Supplement: Supplementary file 2 [file Table_2.docx]

**Supplementary table 2.** Multivariate linear regression analysis of factors associated with Z-scores of MoCA

| Model | Fitting effect | Coefficient *β*(95%CI) | *P* | VIF |
| --- | --- | --- | --- | --- |
| Constant | R=0.592  R^2^=0.351  Adjusted R^2^=0.336 | 0.181(-0.417~0.779) | 0.550 |  |
| Education |  | 0.092(0.056~0.127) | 0.000 | 1.022 |
| PDQ39 |  | -0.015(-0.021~-0.008) | 0.000 | 1.082 |
| CSVD burden |  | -0.224(-0.437~-0.051) | 0.014 | 1.089 |

| Model summary | R | R^2^ | Adjusted R^2^ | F | *P* |
| --- | --- | --- | --- | --- | --- |
| 1 | 0.426 | 0.182 | 0.175 | 29.07 | 0.000 |
| 2 | 0.565 | 0.319 | 0.309 | 30.47 | 0.000 |
| 3 | 0.592 | 0.351 | 0.336 | 23.22 | 0.000 |

Model 1 predictor: Education; Model 2 predictors: Education, PDQ39; Model 3 predictors: Education, PDQ39, CSVD burden.

Abbreviations: MoCA, Montreal Cognitive Assessment; PDQ39, Parkinson’s disease questionnaire 39; CSVD, cerebral small vessel disease; VIF, variance inflation factor.
