# Supplementary material for: Characteristics of Cognitive Impairment and Their Relationship With Total Cerebral Small Vascular Disease Score in Parkinson’s Disease
Source: Front Aging Neurosci. 2022 Jul 7;14:884506. doi: 10.3389/fnagi.2022.884506 (PMC9301002; doi:10.3389/fnagi.2022.884506)
Supplement: Supplementary file 3 [file Table_3.docx]

**Supplementary table 3.** Comparison of Z-scores of MoCA subscores between CSVD = 0 & ≥1 after propensity score matching

|  | CSVD = 0  N=57 | CSVD ≥1  N=50 | *P* |
| --- | --- | --- | --- |
| Total score | 0.27±0.80 | -0.29±1.11 | **0.004** |
| Visuospatial/executive | 0.14±0.97 | -0.16±1.01 | 0.118 |
| Naming | 0.12±0.81 | -0.13±1.18 | 0.213 |
| Attention | 0.07±0.99 | -0.08±1.02 | 0.455 |
| Language | 0.18±0.81 | -0.21±1.14 | **0.040** |
| Abstraction | 0.03±1.00 | -0.03±1.01 | 0.730 |
| Delayed recall | 0.22±0.99 | -0.25±0.96 | **0.014** |
| Orientation | 0.22±0.75 | -0.26±1.17 | **0.015** |

Abbreviations: MoCA, Montreal Cognitive Assessment; CSVD, cerebral small vessel disease.
